# Adjunctive use of *Streptococcus salivarius* M18 probiotic in the treatment of periodontitis: a randomized controlled trial

**DOI:** 10.1007/s13205-025-04363-w

**Published:** 2025-05-28

**Authors:** Wei-Ju Chen, Lavanya Ajay Sharma, Peng Shao, Tia Griffith, Robert Love, Rohit Jain, John Hale, Ajay Sharma

**Affiliations:** 1 School of Medicine and Dentistry, Griffith University, Gold Coast, QLD 4215 Australia; 2 BLIS Technologies, PO Box 2208, South Dunedin, Dunedin, 9044 New Zealand

**Keywords:** *Streptococcus salivarius* M18 probiotic, Periodontitis, Pocket probing depth, Bleeding on probing, Plaque index, Periodontal pathogens

## Abstract

This randomized double-blind placebo-controlled clinical trial aimed to evaluate the adjunctive use of *Streptococcus salivarius* M18 probiotic lozenges in the treatment of periodontitis. Following non-surgical periodontal therapy (NSPT), 55 participants with stage III or IV periodontitis were administered either *S. salivarius* M18 lozenges (test group) or a placebo for 12 weeks. Clinical assessments, including pocket probing depth (PPD), clinical attachment loss (CAL), bleeding on probing (BoP), and plaque index (PI), were performed at baseline (before treatment), immediately after treatment, and during post-treatment follow-ups at 12 and 24 weeks. Microbial analysis was conducted on the subgingival plaque samples collected. The test group demonstrated significantly improved PPD, BoP, and PI compared to the placebo group at post-treatment follow-ups, although no significant difference was observed in CAL. Microbiological analysis revealed a reduction in periodontal pathogens or a shift in the subgingival microbiota toward a decreased pathogenic profile in the test group. This trial is the first to demonstrate the safety and efficacy of *S. salivarius* M18 as an adjunctive treatment for periodontitis, supporting its potential for broader clinical use in managing periodontal health.

**Supplementary Information:**

The online version contains supplementary material available at 10.1007/s13205-025-04363-w.

## Introduction

Periodontitis is considered the sixth most common human disease, affecting 11.2% of the world’s population in its most severe form (Kassebaum et al. 2014). It is an inflammatory disease induced by bacterial plaque that causes host-mediated destruction of the periodontium surrounding and supporting the dentition (Bartold and Van Dyke 2013). During this process, the destruction of collagen fibers in the periodontal ligament and activation of osteoclast cells results in attachment loss and bone resorption (Mehrotra and Singh 2023).

The clinical assessment of periodontitis involves a number of important indicators (Preshaw 2015). Pocket probing depth (PPD) is the distance from the gingival margin to the bottom of the gingival sulcus and represents the apical extension of the inflammatory lesion, while clinical attachment loss (CAL) also considers the presence of gingival recession (REC) and is measured from the cementoenamel junction (CEJ) instead (Lindhe and Lang 2015). Both PPD and CAL are key factors used in the staging of periodontitis (Tonetti et al. 2018). Additionally, bleeding on probing (BoP) is the incidence of bleeding after a thin graduated periodontal probe is inserted into the bottom of the pocket with a light force and indicates the occurrence of subgingival inflammation (Ainamo and Bay 1975). Lastly, plaque index (PI) is the percentage of plaque presence on all the available tooth surfaces and serves as a record of plaque control (O’Leary et al. 1972).

The treatment of periodontitis typically begins with the non-surgical phase that aims to reduce the bacterial load at all tooth sites below the threshold level at which the individual host can manage the remaining infection (Newman et al. 2012; Tomasi et al. 2023). This non-surgical periodontal therapy (NSPT) primarily encompasses plaque control through scaling and root planning, along with patient education via oral hygiene instructions (OHI; Sanz et al. 2020). Clinical endpoints of treatment success may be defined as BoP in < 10% of sites and pocket closure with PPD of ≤ 4 mm (Sanz et al. 2020). Successfully treated patients then enter the maintenance phase involving periodic re-examination, while others may need surgical interventions involving the recontouring of the gingiva or alveolar bone (Newman et al. 2012).

The oral cavity harbors the second largest microbiome in the human body, after the intestine, with various commensal microorganisms (Caselli et al. 2020). Among the over 700 bacterial species colonizing the oral cavity, a subset commonly detected in healthy individuals that includes the *Streptococcus*, *Veillonella*, *Neisseria*, and *Actinomyces* spp. are identified as the “core oral microbiome” (Burcham et al. 2020; Caselli et al. 2020). An imbalance in the host periodontal microbiota, characterized by elevated proportions of pathogenic species, is associated with periodontitis (Hajishengallis and Lamont 2012; Socransky and Haffajee 1992). Therefore, the administration of probiotics to increase the proportion of beneficial bacteria in the oral cavity has been proposed as an adjunct in the prevention and treatment of periodontitis (Hardan et al. 2022). Although most studies on probiotics and periodontal health have been conducted on healthy or gingivitis patients, the adjunctive use of probiotics has shown improvements in multiple parameters, such as PPD, CAL, BoP, and PI, along with reduction in pathogenic bacteria and pro-inflammatory markers (Gatej et al. 2017; Laleman and Teughels 2015). Probiotics may function through competition with pathogens for nutrients, production of antimicrobial substances, and modulation of the host immune system (Ciorba and Stenson 2009).

*Streptococcus salivarius* is a commensal and pioneer colonizer of the human oral cavity present in large populations throughout life (Wescombe et al. 2009). It is a Gram-positive, oxidase-negative, and catalase-negative facultative anaerobic bacterium generally associated with optimal oral health (Poorni et al. 2022). The natural occurrence of *S. salivarius* has been linked with decreased incidences of streptococcal throat infections (Wescombe et al. 2009), and *S. salivarius* lozenges have been marketed to support defense against virulent streptococcal strains (Poorni et al. 2022). *S. salivarius* M18 is an oral commensal strain originally isolated from a healthy adult and is considered an ideal candidate for a model probiotic for the oral cavity (Di Pierro et al. 2015; Wescombe et al. 2009). It produces antimicrobial peptides known as bacteriocin-like inhibitory substance (BLIS) that exhibit interspecies inhibition against similar or related bacteria, including streptococcal pathogens, such as the caries-causing *Streptococcus mutans* (Salim et al. 2023; Wescombe et al. 2006). *S. salivarius* M18 has been assessed in previous randomized controlled clinical trials for reducing caries risk. Burton et al. (2013a) found significantly lower plaque scores in the *S. salivarius* test group compared to the placebo, while Di Pierro et al. (2015) reported a significantly decreased chance of caries development in children using this probiotic. Recently, Salim et al. (2023) demonstrated that the application of *S. salivarius* M18 probiotics significantly decreased *S. mutans* counts, showing a linear correlation between the *S. mutans* counts and caries experience in children.

Although the role of *S. salivarius* as a probiotic in periodontal health has been sparsely studied, its known antimicrobial properties and influence on oral microbiota suggest a strong biological rationale for its potential therapeutic benefits. Current clinical trials in the periodontal field have mainly shown the positive effects of *Lactobacillus* probiotics, particularly *Lactobacillus reuteri* (Hu et al. 2021; İnce et al. 2015; Tekce et al. 2015; Teughels et al. 2013; Vivekananda et al. 2010). Since *Streptococcus* spp. are common colonizers in periodontitis and recolonize the periodontal pocket soon after NSPT (Jünemann et al. 2012), probiotics containing *Streptococcus* spp. may also be beneficial in preventing and treating periodontal disease. Additionally, while supragingival plaque control is crucial for the long-term control of periodontitis (Baker 1995; Hujoel et al. 2005), previous clinical trials have indicated the effectiveness of *S. salivarius* M18 in reducing plaque score and *S. mutans* counts (Burton et al. 2013a; Salim et al. 2023). To date, no clinical trial has specifically evaluated the use of *S. salivarius* in the management of periodontal disease, highlighting a critical gap in the current evidence base that this study aims to address. Thus, this study is designed as the first randomized double-blind placebo-controlled clinical trial to investigate the effectiveness of *S. salivarius-*containing probiotics as an adjunct to NSPT in periodontitis. The objective of this study is to evaluate the clinical and microbiological effects of the adjunctive use of a *S. salivarius* M18-containing lozenge after NSPT for 12 weeks in patients with stage III or IV periodontitis.

## Methods

### Subjects and criteria

**Fig. 1:**
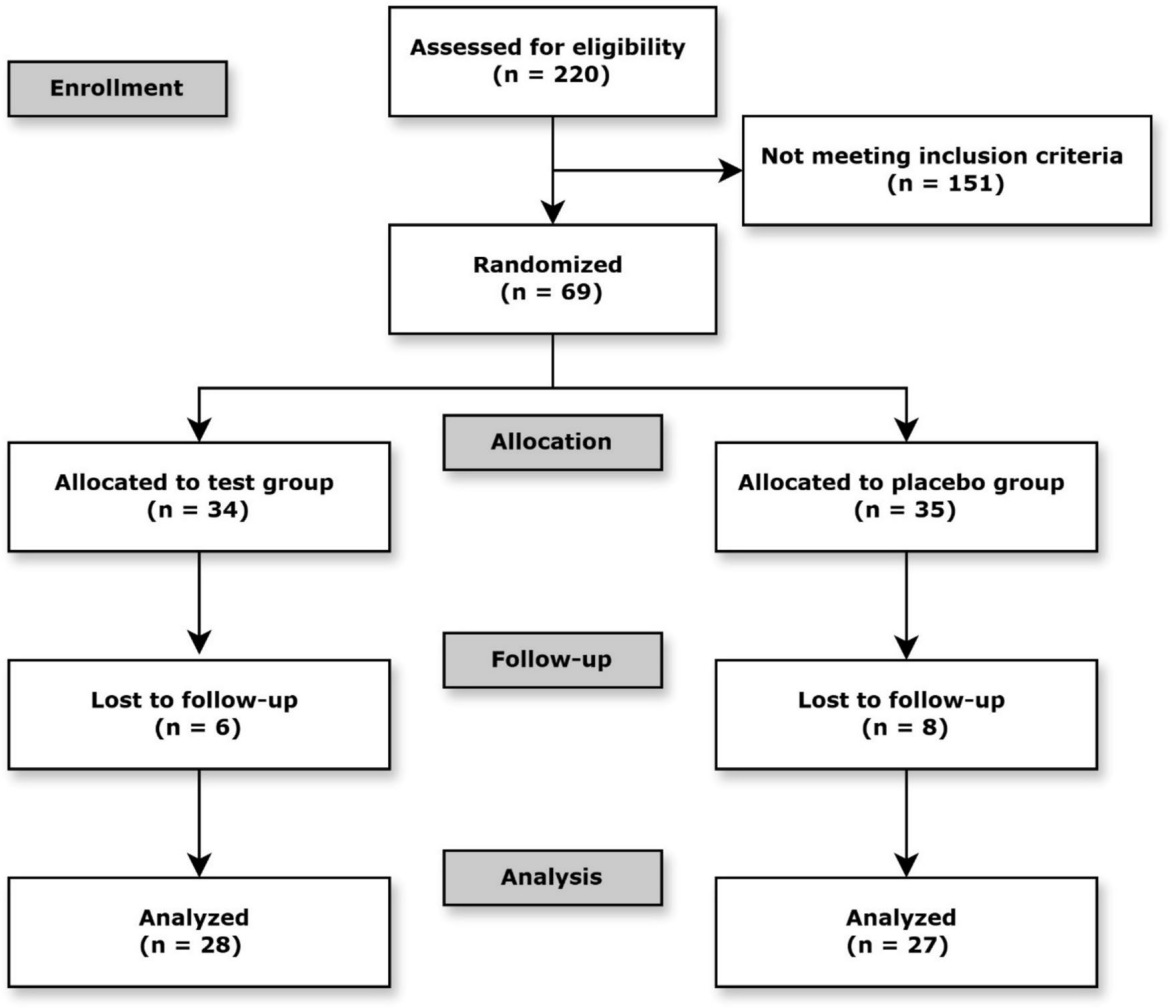
Flow diagram of the study. The diagram illustrates the flow of participants through each phase of the clinical trial, including enrollment, allocation, follow-up, and analysis. A total of 220 participants were assessed for eligibility; 69 met the inclusion criteria and were randomized into two groups. Due to losses to follow-up, 28 participants in the test group and 27 in the placebo group were included in the final analysis

A total of 220 patients who expressed interest in participation were screened according to the inclusion and exclusion criteria listed in Table 1 (Fig. 1). Sixty-nine eligible patients were assigned into two groups using a computer-generated randomization sequence. Test product and placebo lozenges were prepared and labeled according to the randomization results by an individual not involved in the clinical procedures or outcome assessments, ensuring blinding of both participants and investigators. Due to patient dropouts during the follow-up period, 28 participants in the test group and 27 in the placebo group completed the study.

**Table 1:** Inclusion and Exclusion Criteria

Inclusion criteria
1. Systemically healthy (ASA I or II classification)
2. At least 25 years of age
3. A minimum of three natural teeth in every quadrant
4. Untreated stage III or stage IV periodontitis as per the new classification of periodontitis

Exclusion criteria
1. History of diabetes
2. Chronic smoker (> 10 cigarettes/day)
3. Regular antibiotics use in the last six months
4. Regular use of mouthwash in the last six months
5. Other systemic conditions affecting periodontal tissue (e.g., rheumatic fever, liver or kidney disease, immunocompromised conditions)
6. Use of medication that may affect periodontal tissue (e.g., phenytoin, cyclosporin, nifedipine, chronic use of nonsteroidal anti-inflammatory drugs)
7. Pregnancy or lactation
8. Acute oral/periodontal lesion or infection
9. Periodontal therapy, including non-surgical periodontal therapy in the last 12 months
10. History of allergies
11. Requiremen**t** for antibiotic prophylaxis for periodontal clinical examination

### Study scheme

This clinical trial was approved by the Human Research Ethics Committee of Griffith University, Australia (GU Ref No:2019/896). All clinical data recording and manipulations were performed between March 2020 and September 2024. Patients who were pre-screened to require periodontal management and satisfied the inclusion and exclusion criteria were invited to participate in the study at the Griffith University Dental Clinic (Gold Coast, Australia). Written consent was obtained from all participants after a thorough explanation of the trial’s design, objectives, and possible side effects and benefits.

Participants were assigned to either the NSPT and *S. salivarius* M18 group or and the NSPT and placebo group through simple randomization. Coded lozenge packs were provided by the study coordinator to the examiner. All study personnel and patients were blinded to group assignments, except for the study coordinator. The identities of the two groups were revealed only after completion of statistical analysis.

### Tested product and placebo

The probiotic lozenge used in this study was BLIS M18™ (>250 million cfu/lozenge at the end of shelf life, BLIS ToothGuard,) (BLIS Technologies, Dunedin, New Zealand). BLIS M18™ contains a freeze-dried culture of *S. salivarius* M18 in a protective matrix. The study product was compared to placebo lozenges without *S. salivarius* M18, identical in appearance, texture, and taste, provided by the manufacturer.

### Treatment protocol

The timeline of the study is summarized in Table 2. Briefly, clinical assessments of periodontitis, including PPD, CAL, BoP, and PI, were performed before treatment (time point P-2), at 12 weeks after the commencement of the test product or the end of the treatment (time point P0), at 12 weeks post-treatment (time point P+2), and at 24 weeks post-treatment (time point P+3). Plaque samples were collected for microbial analysis at time points P-2, P0, and P+2.

**Table 2:** The timeline of the study

Phase	Appointment	Time Point		Recording of Periodontitis Indicators	Collection of Plaque Samples
Pre-treatment	1	P-2	Before treatment	Yes	Yes
Treatment	2		NSPT and commencement of the test product	No	No
	3	P-1	6 weeks after the commencement of treatment	No	No
Post-treatment	4	P0	12 weeks after commencement of treatment; end of treatment	Yes	Yes
	5	P+1	6 weeks after the end of treatment	No	No
	6	P+2	12 weeks after the end of treatment	Yes	Yes
	7	P+3	24 weeks after the end of treatment	Yes	No

A comprehensive baseline periodontal examination was performed before treatment. This examination included full periodontal charting, with PPD, BOP, and REC recorded at six sites per tooth, and PI recorded at four surfaces per tooth. A North Carolina periodontal probe (Hu-Friedy Manufacturing, Chicago, IL, USA) was used for the measurements.

All participants received OHI and were provided with oral hygiene packs containing toothpaste (Colgate Total Original Toothpaste; Colgate-Palmolive, Sydney, Australia), a toothbrush (Colgate Total Professional Ultra Compact Toothbrush; Colgate-Palmolive, Sydney, Australia), floss thread (Colgate Total; Colgate-Palmolive, Sydney, Australia), and interdental brushes (Piksters; Erskine Dental, Macksville, Australia) for use throughout the study.

Initial periodontal therapy with full-mouth NSPT was completed in one or two appointments within two weeks using ultrasonic scalers and hand instruments under local anesthesia. The participants were asked to rinse with 0.2% w/v chlorhexidine mouthwash (Colgate Savacol; Colgate-Palmolive, Sydney, Australia) for two minutes after the completion of NSPT. All clinical manipulations were performed by one Doctor of Clinical Dentistry post-graduate student in Periodontology under the supervision of a periodontist. During the same appointment, participants were randomized to receive either the test product or a placebo lozenge. They were instructed to take one lozenge in the morning and another at night following toothbrushing for a total of 12 weeks. Additionally, participants were asked to refrain from using any other probiotic supplements during the study period. Compliance was monitored by inquiring about the number of lozenges consumed at follow-up visits. A questionnaire (Fig. S1) was given to the patient at P0 to check for any adverse events.

Follow-up visits for review were scheduled at four time points post-treatment: P0, P+1, P+2, and P+3 (Table 2). At each visit, periodontal measurements of PPD, BOP, REC and PI were recorded. Participants were also questioned about any changes in their general health, use of anti-inflammatory medications, mouthrinses, or other probiotic products.

### Clinical indicators of periodontitis

Clinical indicators of periodontitis were assessed at four time points: P-2, P0, P+2, and P+3. The following clinical parameters were recorded:

1. PPD: measured in millimeters (mm) at six sites per tooth, excluding third molars.
2. REC: measured in mm at six sites per tooth, recorded as positive when the gingival margin is apical to the CEJ, and negative when coronal.
3. BoP: recorded as presence or absence of bleeding within 10 seconds at six sites per tooth.
4. PI: recorded as presence or absence of plaque at four surfaces per tooth.

PPD was converted to a percentage, representing the proportion of total measured sites across all present teeth with depths greater than 4 mm. CAL was calculated by summing PPD and REC at each site. BOP and PI were expressed as percentages of total sites showing presence of bleeding or plaque, respectively. To assess changes over time, values recorded at P0, P+2, and P+3 were normalized to each participant’s baseline (P-2) values and presented as percentages relative to baseline. PPD was designated as the primary outcome variable, while CAL, BOP, and PI were considered secondary outcome variables.

### Microbiological analysis

At time points P-2, P0, and P+2, subgingival plaque samples were collected from the pocket of the deepest baseline PPD of each participant. The site was first isolated from saliva using cotton rolls and gently dried with compressed air to avoid contamination. Gracey curettes (Hu-Friedy Manufacturing) were used for sample collection. Each sample was dispersed in 0.75 ml of Phosphate Buffered Saline (Gibco, Waltham, MA, USA), mixed by vortex, and immediately stored at − 20 °C. Due to budget constraints, DNA extraction was performed on samples from 15 randomly selected participants in each group using the QIAamp DNA Mini Kit (QIAGEN, Hilden, Germany), following the manufacturer’s instructions. The V4 region of the 16S rDNA of sample DNA was PCR amplified with barcoded primers targeting base positions CAGRF22029560 (for 16S: 27 F—519R) and CAGRF22029561 (for 16S: 341 F—806R). The PCR products were sequenced at the Australian Genome Research Facility (AGRF; Melbourne, Australia).

Raw sequencing data were processed to generate operational taxonomic units (OTUs), and taxonomic assignment was conducted using a reference database. The microbial abundance data were normalized using relative abundance (percentage of each taxon out of total reads per sample) to account for variability in sequencing depth. The relative abundance of bacterial classes was averaged across individuals in each group, and comparisons were performed between the test and placebo groups at each time point (P0 and P+2). Only classes with a mean relative abundance above 1% in at least one group were reported to ensure interpretability and reduce noise from low-abundance taxa.

### Statistical analysis

The minimum number of subjects required for adequate power was determined using an online calculator (https://clincalc.com/stats/samplesize.aspx) with a continuous endpoint for two independent study groups. It was calculated that 26 participants were required in each group to provide 95% power with an alpha of 0.05.

Descriptive statistics was first performed for demographic variables. Participant age and gender were compared across the two groups using a t-test and Fisher’s exact test, respectively. The clinical variables, namely PPD, CAL, BOP, and PI, at time points P0, P+2, and P+3 were first normalized to the baseline values at P-2. Their differences between the NSPT and *S. salivarius* M18 group and the NSPT and placebo group at each time point and the differences among the different time points within each group were compared using two-way mixed-model ANOVA with Geisser–Greenhouse correction followed by Tukey’s multiple comparisons test. Two-way mixed-model ANOVA allows for the assessment of both within-subject (time) and between-subject (treatment group) effects simultaneously (Keppel and Wickens 2004), while Geisser-Greenhouse correction accounts for violations of sphericity (Geisser and Greenhouse 1958). All statistical analyses were performed using Prism 10.4.1 (GraphPad Software, San Diego, CA, USA) with a significance threshold of p ≤ 0.05.

## Results

### Participant demographics

A total of 220 patients were screened, and 69 patients were eligible for the study. Twenty-eight participants in the test group and 27 participants in the placebo group completed the study and were included in the analysis of clinical data. The mean ages of participants in the test group and the placebo group were 59.6 (± 10.4) and 60.1 (± 12.1), respectively. The test group included 20 males and 8 females, whereas the placebo group included 12 males and 15 females. No significant difference in age and gender was found between the two groups.

No compliance issue was noted. No adverse effect of the test product was reported by the participants or observed by the investigators, except for one patient who experienced constipation. *S. salivarius* M18 in the lozenge remained stable throughout the trial and beyond, with a decrease of less than 0.1 log in cell count at 36 months compared to the baseline.

### Clinical indicators of periodontitis

For each patient, the percentages of sites with PPD > 4 mm, the calculated values of CAL in mm, the percentages of sites with BoP, and the percentages of sites with plaque at time points P0, P+2, and P+3 were normalized to the percentages of the baseline at time point P-2. For the NSPT and *S. salivarius* M18 group at time points P-2, P0, P+2, and P+3, the PPD > 4 mm was 100%, 46.14% (± 22.08%), 32.33% (± 21.07%), and 24.86% (± 16.84%), respectively; the CAL was 100%, 93.99% (± 12.13%), 90.96% (± 12.14%), and 89.67% (± 13.40%), respectively; the BoP was 100%, 36.50% (± 14.50%), 26.13% (± 11.54%), and 19.04% (± 9.60%), respectively; the PI was 100%, 35.17% (± 14.74%), 24.62% (± 15.75%), and 15.94% (± 9.75%), respectively (Figure S2). For the NSPT and placebo group at time points P-2, P0, P+2, and P+3, the PPD > 4 mm was 100%, 61.60% (± 25.46%), 43.85% (± 21.69%), and 29.46% (± 15.13%), respectively; the CAL was 100%, 91.90% (± 11.30%), 90.46% (± 11.96%), and 87.29% (± 11.55%), respectively; the BoP was 100%, 51.04% (± 24.61%), 37.83% (± 13.44%), and 25.08% (± 10.94%), respectively; the PI was 100%, 50.86% (± 21.06%), 37.90% (± 23.53%), and 23.23% (± 16.94%), respectively.

As expected, the PPD > 4 mm, BoP, and PI of both the test group and the placebo group were significantly reduced at P0, P+2, and P+3 as compared to the P-2 baseline (*P* ≤ 0.0001). For the CAL, significant reductions were observed in the test group at P+2 (*P* = 0.0027) and P+3 (*P* = 0.0019) as compared to the P-2 baseline, and in the placebo group at P0 (*P* = 0.0049), P+2 (*P* = 0.0017), and P+3 (*P* ≤ 0.0001); at P0 in the test group, the reduction of CAL was nearly statistically significant (*P* = 0.0640). Figure S2 indicates the levels of significance of the pairwise comparisons at different time points within each group.

**Fig. 2:**
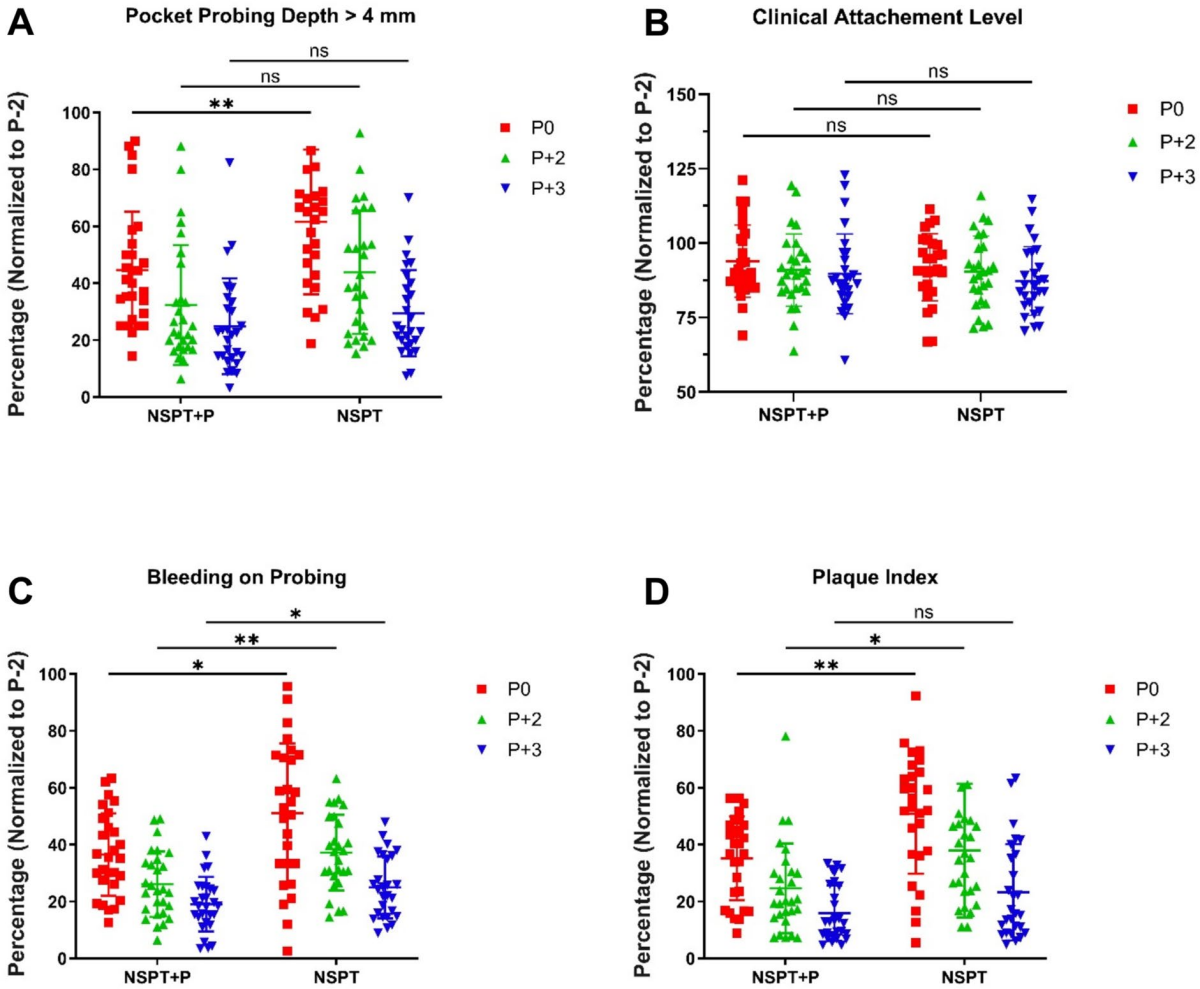
Clinical indicators of periodontitis in the NSPT and *S. salivarius* M18 group (NSPT+P) and the NSPT and placebo group (NSPT) at the time points P0 (end of the 12-week treatment), P+2 (12 weeks after the end of treatment), and P+3 (24 weeks after the end of treatment), presented in percentages normalized to the baseline at the time point P-2 and compared between the two groups at each time point. **A** Pocket probing depth greater than 4 mm (PPD > 4 mm). The *S. salivarius* M18 treatment significantly reduced the PPD > 4 mm at P0. **B** Clinical attachment loss. **C** Bleeding on probing (BoP). The *S. salivarius* M18 treatment significantly reduced the BoP at P0, P+2, and P+3. **D** Plaque index (PI). The *S. salivarius* M18 treatment significantly reduced the PI at P0 and P+ 2. (***P ≤ 0.001, **P ≤ 0.01, *P ≤ 0.05; two-way mixed-model ANOVA, Tukey’s multiple comparisons test)

Regarding the main objective of this study, the clinical indicators at each time point were compared between the test group and the placebo group (Fig. 2). The PPD > 4 mm, BoP, and PI were all significantly lower in the test group than the placebo at P0. At P0, the *S. salivarius* M18 treatment significantly reduced the PPD > 4 mm from 61.60% (± 25.46%) to 46.14% (± 22.08%; *P* = 0.0090), significantly reduced the BoP from 51.04% (± 24.61%) to 36.50% (± 14.50%; *P* = 0.0111), and significantly reduced the PI from 50.86% (± 21.06%) to 35.17% (± 14.74%; *P* = 0.0025). The reduction in PPD > 4 mm associated with the probiotic treatment was nearly statistically significant at P+2 (*P* = 0.0510). The reduction in BoP was also statistically significant at P+2 (*P* = 0.0019) and P+3 (*P* = 0.374) in addition to P0. Regarding PI, the reduction in the treatment group was statistically significant at P+2 (*P* = 0.0182) and nearly statistically significant at P+3 (*P* = 0.0585). No significant difference in the CAL was observed between the two groups at any time point.

### Microbiological analysis

**Fig. 3:**
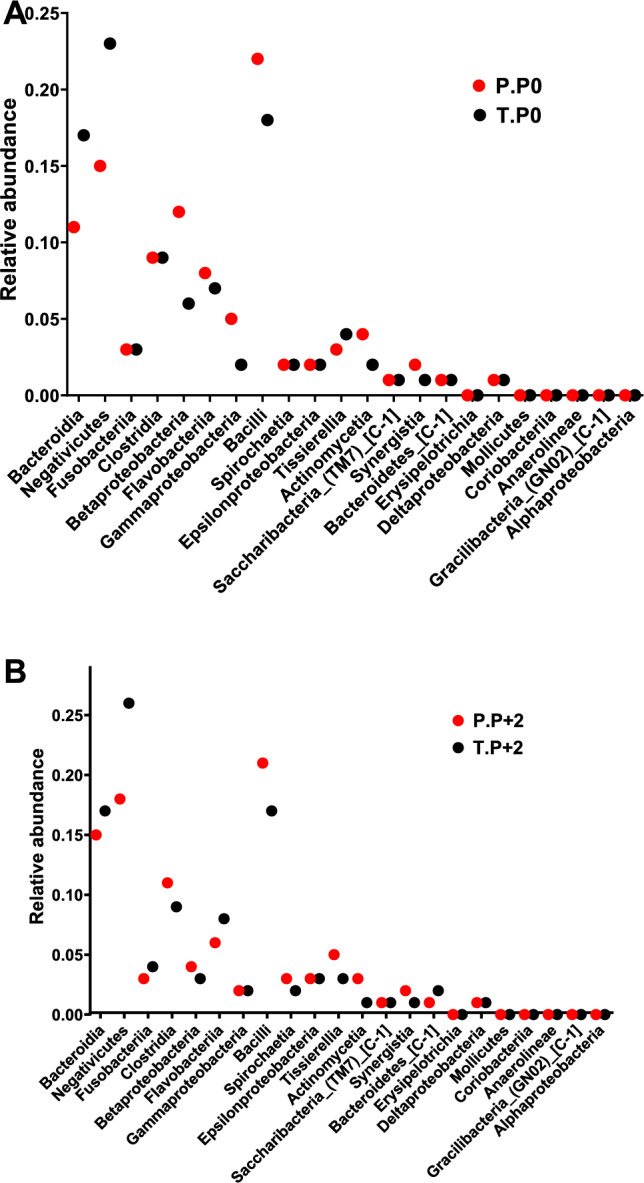
Microbial analysis of 22 bacterial classes from the placebo (P) and test (T) groups at two time points: **A** end of treatment (P0) and **B** 12 weeks post-treatment (P+2). At P0, the test group exhibited a lower relative abundance of *Betaproteobacteria*, *Flavobacteriia*, *Gammaproteobacteria*, *Bacilli*, and *Actinomycetia* compared to the placebo group. By P+2, further reductions were observed in *Clostridia*, *Betaproteobacteria*, *Bacilli*, *Spirochaetia*, *Tissierellia*, *Actinomycetia*, and *Synergistia* in the test group

The microbiological analysis demonstrated notable changes in the microbial composition of subgingival plaque following the adjunctive use of *S. salivarius* M18 probiotic lozenges in the test group compared to the placebo group. At the treatment endpoint (P0), the abundance of microbial classes, such as *Betaproteobacteria*, *Flavobacteriia*, *Gammaproteobacteria*, *Bacilli*, and *Actinomycetia*, was decreased in the test group compared to the placebo group (Fig. 3A). However, other microbial classes, including *Fusobacteriia*, *Clostridia*, *Spirochaetia*, *Epsilonproteobacteria*, *Saccharibacteria*_(TM7)_[C-1], *Bacteroidetes*_[C-1], *Erysipelotrichia*, *Deltaproteobacteria*, *Mollicutes*, *Coriobacteriia*, *Anaerolineae*, and *Gracilibacteria*_(GN02)_[C-1], showed similar abundance in both groups at this time point.

At 12 weeks post-treatment (P+2), the abundance of *Clostridia*, *Betaproteobacteria*, *Bacilli*, *Spirochaetia*, *Tissierellia*, *Actinomycetia*, and *Synergistia* was reduced in the test group compared to the placebo group. In contrast, microbial classes, such as *Gammaproteobacteria*, *Epsilonproteobacteria*, *Saccharibacteria*_(TM7)_[C-1], *Erysipelotrichia*, *Deltaproteobacteria*, *Mollicutes*, *Coriobacteriia*, *Anaerolineae*, *Gracilibacteria*_(GN02)_[C-1], and *Alphaproteobacteria*, were similar in both groups at P+2 (Fig. 3B).

## Discussion

This study presents the first randomized double-blind placebo-controlled clinical trial of the adjunctive use of *S. salivarius* M18-containing lozenges for the treatment of periodontitis. The test product was administered to the participants who were diagnosed with stage III or IV periodontitis for 12 weeks twice daily immediately after NSPT. The clinical parameters, including PPD, CAL, BoP, and PI, were assessed and the plaque samples were collected during the 24-week follow-up period after the end of the 12-week treatment. Clinical data and microbiological analysis support the effectiveness of *S. salivarius* M18 as an adjunct to NSPT in the treatment of periodontitis. Meanwhile, the results also attested to the safety of this product as only one participant reported minor gastrointestinal upset in the questionnaire-based survey of adverse effects. The dosing regime and the formulation of this product were also found to be suitable for stage III or IV periodontitis patients since no compliance issue was reported.

As the primary outcome variable, the number of sites with PPD > 4 mm normalized to the baseline was significantly reduced in the test group compared to the placebo at the completion of the 12-week treatment (P0) and remained nearly significantly reduced at 12 weeks after the completion of the 12-week treatment (P+2; Fig. 2A). At 24 weeks after the completion of the 12-week treatment (P+3), there was still a non-significant trend of decreased PPD > 4 mm sites in the test group. Since PPD is accepted as a parameter to determine the need for periodontal surgery, the adjunctive use of *S. salivarius* M18-containing test product may effectively help periodontitis patients enter the maintenance phase with periodic re-examination instead of the invasive and costly surgical phase after the completion of the initial NSPT (Sanz et al. 2020).

Similarly, the secondary outcome variables, the BoP and PI normalized to the baseline, were both significantly reduced in the test group than the placebo at the completion (P0) and at 12 weeks after the completion of the 12-week treatment (P+2; Fig. 2C, D). At 24 weeks after the treatment (P+3), the reduction in BoP remained statistically significant and the reduction in PI was nearly statistically significant. Plaque is the primary etiological agent in gingival and periodontal inflammation (Loe et al. 1965). Since gingivitis and periodontitis are inflammatory responses initiated by oral pathogens that colonize to form a plaque biofilm (Bartold and Van Dyke 2013), the reductions in plaque levels indicated by PI and in inflammation levels indicated by BoP achieved in this clinical trial support the effectiveness of the *S. salivarius* M18-containing test product in attaining and maintaining periodontal health.

Although significant improvements in PPD, BoP, and PI were observed in the treatment group, no statistically significant difference in CAL was found between the test and the placebo groups (Fig. 2B). This aligns with the established understanding that CAL reflects irreversible loss of periodontal support, which can only be regenerated through surgical procedures, not by NSPT alone (Newman et al. 2012). This is supported by our clinical results of the placebo group (Fig. S2B). Here, even when supplemented with *S. salivarius* M18, NSPT remains limited in its ability to regenerate lost attachment. Therefore, *S. salivarius* M18 may be viewed as a tool for disease stabilization and prevention of further attachment loss, rather than for reversal of existing tissue destruction. Future long-term studies are needed to evaluate whether probiotic use can contribute to maintaining CAL stability or enhance regenerative outcomes when combined with surgical periodontal therapies.

In addition to comparisons between the test and placebo groups at various time points, intra-group comparisons over time are also noteworthy. Significant reductions in PPD > 4 mm, BoP, and PI were observed progressively at each time point in both groups, reflecting the effectiveness of standard NSPT in managing periodontitis (Figure S2). Importantly, the test group showed significantly greater reductions in these parameters compared to the placebo group, which had already benefited from NSPT alone (Fig. 2). This highlights the added value of *S. salivarius* M18 as an adjunctive treatment. However, given that NSPT alone achieved substantial clinical improvements, the limited room for further reduction may at least partially explain the continued downward trend in PPD > 4 mm without reaching statistical significance at P+2 and P+3.

Meanwhile, the microbiological analysis revealed significant alterations in the microbial composition of subgingival plaque in the test group compared to the placebo group, highlighting the potential role of *S. salivarius* M18 in modulating periodontal microbiota and improving clinical outcomes. At the treatment endpoint (P0), a reduction in the relative abundance of *Actinomycetia*, *Flavobacteriia*, and *Gammaproteobacteria* was observed in the test group. Multiple *Actinomycetia* spp. have been associated with periodontitis (Vielkind et al. 2015). *Flavobacteriia* have also been reported in higher proportion in periodontal disease (López-Martínez et al. 2020). *Aggregatibacter actinomycetemcomitans*, a member of the *Gammaproteobacteria* class, is a recognized periodontal pathogen with multiple virulence factors (Belibasakis et al. 2019). Furthermore, at 12 weeks post-treatment (P + 2), reductions were also noted in *Clostridia*, *Spirochaetia*, *Synergistia*, and *Tissierellia*, in addition to the aforementioned *Actinomycetia*. *Clostridia*, *Spirochaetia*, and *Synergistia* have all been found to be more abundant in periodontitis (Abusleme et al. 2013; Cai et al. 2021; Griffen et al. 2012; López-Martínez et al. 2020; Yousefi et al. 2020). Among them, *Treponema denticola*, a highly pathogenic spirochete, was recognized early on as one of the three species comprising the “red complex” associated with periodontitis (Socransky et al. 1998; Yousefi et al. 2020). Additionally, *Filifactor alocis*, a member of the *Clostridia* class, has been extensively investigated for its involvement in the pathogenesis of periodontitis for the past decade (Aruni et al. 2015; Manenzhe et al. 2024). These findings suggest that *S. salivarius* M18 may exert beneficial microbiological modulation by reducing key bacterial taxa implicated in periodontal disease. The resulting reduction in dysbiosis likely diminishes host immune activation, thereby contributing to clinical improvements, such as PPD > 4 mm and BoP. The delayed reduction in certain highly pathogenic anaerobes at 12 weeks post-treatment (P+2) suggests that sustained probiotic colonization may be necessary to achieve optimal microbial rebalancing. Overall, these microbiological changes support the hypothesis that restoring microbial homeostasis through probiotic intervention can reduce periodontal inflammation and promote tissue healing. The microbial shifts observed in our study provide a mechanistic explanation for the clinical improvements seen in the test group receiving *S. salivarius* M18.

As the first clinical trial examining the adjunctive use of *S. salivarius* M18 for the treatment of periodontitis, no previous data are available for direct comparison. However, Laleman et al. (2015) presented the only available randomized placebo-controlled clinical trial to our knowledge on the effectiveness of *Streptococcus* spp. alone in the treatment of NSPT. A tablet containing *Streptococcus oralis* KJ3, *Streptococcus uberis* KJ2, and *Streptococcus rattus* JH145 probiotics was administered after NSPT twice daily for 12 weeks. Clinical indicators, including PPD, REC, CAL, BoP, gingival index (GI), and PI, were measured and microbiological analysis was performed on plaque samples collected at multiple time points. As no significant difference was found between the test and the placebo groups except for the improved PI in the test group at 24 weeks, it was concluded that their test product of *Streptococcus* probiotics demonstrated no clinical or microbiological benefits in the treatment of periodontitis. The positive results in contrast of this present study on *S. salivarius* M18 with a similar experimental design indicates the species specificity of the therapeutic effects of probiotic supplements on periodontitis within the *Streptococcus* genus. Since this difference may also be attributable to other factors, such as strain, dosage, and vehicle, it may not be conclusive that *S. salivarius* M18 has better performance than *S. oralis* KJ3, *S. uberis* KJ2, or *S. rattus* JH145. Nonetheless, it is clear that the presented *S. salivarius* M18-containing lozenge with the given regime and formulation in our study established the most superior results of *Streptococcus* spp. in the adjunctive treatment of periodontitis in the existing literature, achieving significant improvements in both clinical indicators and microbiological compositions.

Similarly, being the first trial of its kind, this study demonstrated for the first time the efficacy of *S. salivarius* M18 probiotics in modulating subgingival microbial communities. The results are consistent with available studies which focused on salivary bacteria and supragingival plaque. For instance, Burton et al. (2013a) demonstrated that treatment with *S. salivarius* M18 significantly reduced plaque scores in children, particularly in those with high initial plaque levels; meanwhile, the subgroup of subjects who were the most effectively colonized with *S. salivarius* M18 exhibited reduced *S. mutans* counts in saliva. Furthermore, Di Pierro et al. (2015) showed that *S. salivarius* M18 increased the likelihood of avoiding new dental caries development by 1.9-fold, supporting the effectiveness of *S. salivarius* M18 to counteract dental plaque inhabitants. Although our study focused on subgingival plaque rather than salivary or supragingival microorganisms, the reduction in *Bacilli*, which encompasses plaque-associated genera, aligns with these results. The reduction in the previously discussed microbial classes associated with periodontitis in our study, including *Actinomycetia*, *Clostridia*, *Flavobacteriia*, *Gammaproteobacteria*, *Spirochaetia*, *Synergistia*, and *Tissierellia*, provides further evidence of its potential for improving periodontal health. The clinical implications of this study are significant, as periodontitis is driven by a dysbiotic microbial community that contributes to inflammation and tissue destruction. The observed shifts in microbial composition suggest that *S. salivarius* M18 may help restore microbial balance in the subgingival environment, thereby mitigating the pathogenic processes underlying periodontitis. These findings highlight the potential of *S. salivarius* M18 as a novel adjunctive therapy for managing periodontitis, particularly in patients with high plaque levels or poor periodontal outcomes.

Current evidence suggests that the improved periodontal health observed with *S. salivarius* M18 treatment may result from both the suppression of pathogenic bacteria and the modulation of host immune responses. The strain has demonstrated inhibitory activity against major periodontal pathogens, including *Porphyromonas gingivalis*, *T. denticola*, *Tannerella forsythia*, and *Fusobacterium nucleatum* (Burton et al. 2013b; Park et al. 2023). A key mechanism underlying this antagonism is the production of BLIS, such as salivaricin A2 and salivaricin B, which exhibit antimicrobial properties (Burton et al. 2013b; Salim et al. 2023; Wescombe et al. 2006). Moreover, *S. salivarius* M18 expresses urease, enabling the hydrolysis of urea into ammonia, thereby increasing the local pH and creating an environment less favorable to acidogenic and pathogenic bacteria (Di Pierro et al. 2015; Salim et al. 2023). The strain also produces dextranase, which degrades the dextran matrix that contributes to dental plaque formation (Di Pierro et al. 2015; Salim et al. 2023). Furthermore, as a proficient colonizer of the oral cavity, *S. salivarius* M18 may compete with pathogenic bacteria for adhesion sites and disrupt bacterial coaggregation and attachment dynamics (Burton et al. 2013b; Salim et al. 2023). Finally, it exhibits immunomodulatory properties by attenuating inflammatory responses, notably through the downregulation of pro-inflammatory cytokines, such as interleukin 6 and interleukin 8, induced by periodontal pathogens (MacDonald et al. 2021).

In the field of periodontitis management, *L. reuteri* and other *Lactobacillus* spp. have been the most investigated probiotics with promising results (Hu et al. 2021). Multiple clinical trials have generally shown significantly improved PI, BoP, PPD, and CAL with adjunctive use of *L. reuteri*-containing lozenges (İnce et al. 2015; Tekce et al. 2015; Teughels et al. 2013; Vivekananda et al. 2010). In our study, PI, BoP, and PPD all had significant improvement with the test product containing *S. salivarius* M18. The absence of statistical significance in CAL may largely be attributed to the variability of the experimental designs. The therapeutic effects of *S. salivarius* M18 and *L. reuteri* cannot be confidently compared without another clinical trial directly comparing the two species.

In addition to the adjunctive use of *S. salivarius* M18 alone in periodontitis, it may also be used in combinations. Mani et al. (2017) showed the positive clinical results of a *S. salivarius*, *L. reuteri*, and *Lactobacillus paracasei* blend. Due to the presence of the two *Lactobacillus* spp., the role of *S. salivarius* in the combination was not understood. Being the first clinical trial to confirm its therapeutic effects, this present study provides direct evidence for the future investigation and development of probiotic combinations containing *S. salivarius* M18.

While the study highlights the clinical and microbiological benefits of *S. salivarius* M18, there are limitations that should be acknowledged. First, the sample size for microbiological analysis was limited to 15 participants per group due to resource constraints. This may reduce statistical power, increase the influence of interindividual variability, and limit the generalizability of the results. Second, the duration of the treatment was limited to 12 weeks. While this timeframe allowed for the assessment of short-term clinical and microbiological effects, it may not capture the long-term impacts of the *S. salivarius* M18 treatment on periodontitis, given that it is a chronic condition. Additionally, although changes in microbial composition were observed, the functional implications of these changes on clinical outcomes warrant further investigation. Future research should include larger cohorts, longer treatment and follow-up periods, and mechanistic studies to confirm and expand upon these results. Moreover, the development of methods to optimize colonization efficiency may be important in enhancing the clinical efficacy of the probiotic (Burton et al. 2013a).

This study provides compelling evidence for the adjunctive use of *S. salivarius* M18-containing lozenges in the treatment of periodontitis. The results demonstrated significant improvements in key clinical parameters, including PPD, BoP, and PI, in the treatment group compared to the placebo. These improvements suggest that the probiotic has a positive effect on periodontal health, particularly in reducing inflammation and plaque levels, which are critical factors in the pathogenesis and progression of periodontitis. These clinical findings were supported by microbiological analysis which revealed a shift in the subgingival microbiota toward a decreased pathogenic profile in the treatment group. This suggests that *S. salivarius* M18 may help restore microbial balance in periodontal tissues, potentially moderating further disease progression. Although no significant difference in CAL was observed, the improvements in PPD, BoP, and PI indicate the potential for *S. salivarius* M18 to support the maintenance phase of periodontal therapy, reducing the need for invasive treatments such as surgeries. Overall, this study confirms that *S. salivarius* M18 is a safe and effective adjunct to NSPT, providing clinical and microbiological benefits that may enhance the management of periodontitis. Given the positive outcomes observed, this probiotic lozenge could offer a valuable non-invasive alternative for patients with periodontitis, especially those with high plaque levels or poor periodontal outcomes. These results may also inform the design of future long-term clinical trials and contribute to the development of evidence-based treatment guidelines incorporating probiotics in periodontal therapy. Future studies with increased sample sizes and prolonged treatment and follow-up periods would be valuable to confirm the long-term effects and further explore the potential of *S. salivarius* M18 in periodontal health.

## Conclusions

This randomized double-blind placebo-controlled clinical trial is the first to demonstrate the safety and efficacy of a *S. salivarius* M18-containing lozenge as an adjunctive treatment of periodontitis. No major adverse effect or compliance issue was reported, confirming its safety and tolerability. The clinical parameters, including PPD, PI, and BoP, showed significantly greater improvement in the test group compared to the placebo group. Additionally, microbiological analysis revealed significant and sustained shifts in the composition of subgingival microbiota, with reductions in pathogenic bacterial classes associated with periodontitis.

These findings provide the first clinical validation of the theoretical rationale based on literature that *S. salivarius* M18 may promote periodontal health by modulating the oral microbiome. The results support its role as an effective adjunct to NSPT and suggest a non-invasive probiotic strategy for enhancing treatment outcomes and maintaining long-term oral health. Incorporating *S. salivarius* M18 probiotics into routine periodontal care could represent a valuable step forward in microbiome-based therapeutic approaches to managing periodontitis.

## Supplementary Information

Below is the link to the electronic supplementary material.

Supplementary file1 (DOCX 704 KB)

## Data Availability

The anonymized individual participant data (IPD) that support the findings of this study are available from the corresponding author upon reasonable request. Access will be granted after approval by an appropriate ethics committee and completion of a data sharing agreement.
