# Correction: Adjunctive use of *Streptococcus salivarius* M18 probiotic in the treatment of periodontitis: a randomized controlled trial

**DOI:** 10.1007/s13205-026-04700-7

**Published:** 2026-01-20

**Authors:** Wei-Ju Chen, Lavanya Ajay Sharma, Peng Shao, Tia Griffith, Robert Love, Rohit Jain, John Hale, Ajay Sharma

**Affiliations:** 1 School of Medicine and Dentistry, Griffith University, Gold Coast, QLD 4215 Australia; 2 BLIS Technologies, PO Box 2208, South Dunedin, Dunedin, 9044 New Zealand

**Correction: 3 Biotech (2025) 15:192**

10.1007/s13205-025-04363-w

In this article on page 3, in section "Tested product and placebo", the sentence beginning "The probiotic lozenge used in this study was BLIS M18™...in this article, the text.

The probiotic lozenge used in this study was BLIS M18™ (BLIS Technologies, Dunedin, New Zealand) should have read as.

The probiotic lozenge used in this study was BLIS M18™ (>250 million cfu/lozenge at the end of shelf life, BLIS ToothGuard,).

The original article has been updated.

