# Supplementary material for: Adjunctive use of Streptococcus salivarius M18 probiotic in the treatment of periodontitis: a randomized controlled trial
Source: 3 Biotech. 2025 May 28;15(6):192. doi: 10.1007/s13205-025-04363-w (PMC12119401; doi:10.1007/s13205-025-04363-w)
Supplement: Supplementary file 1 — Supplementary file1 (DOCX 704 KB) [file 13205_2025_4363_MOESM1_ESM.docx]

**Supplementary**


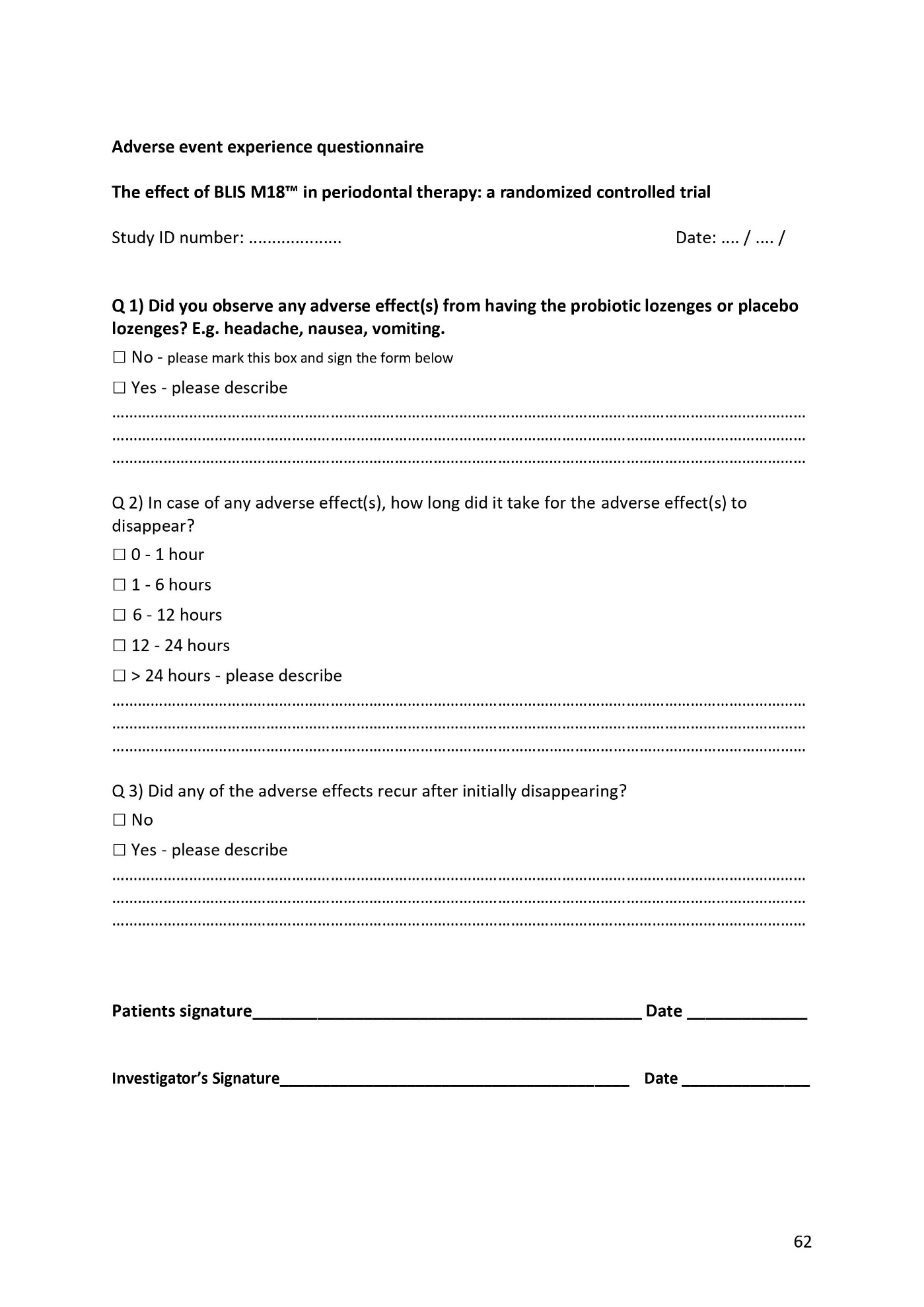


**Supplementary Figure 1.** Questionnaire of adverse event experience completed by participants at the end of the test product treatment (P0).


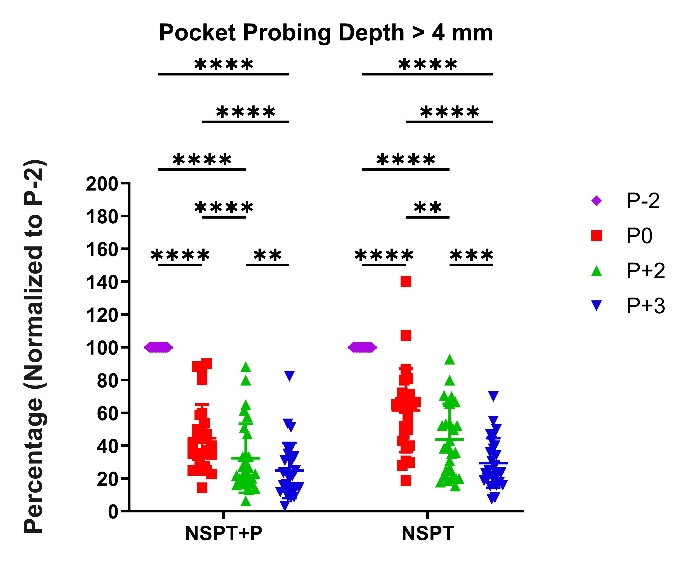

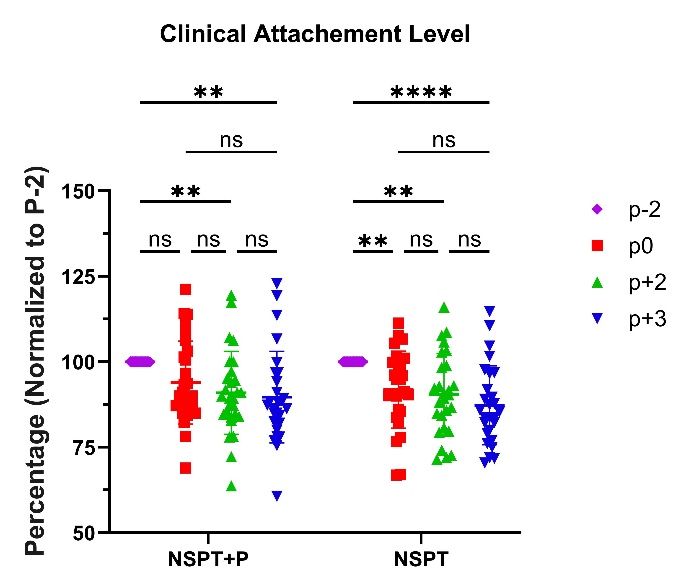


**B**

**A**


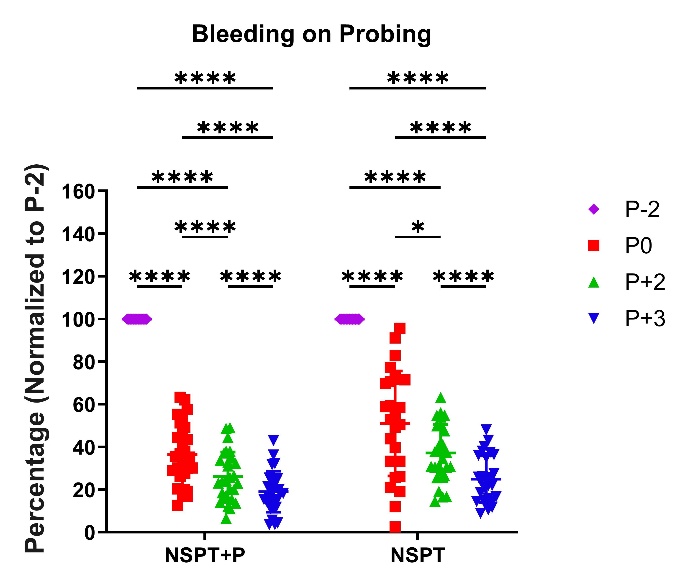

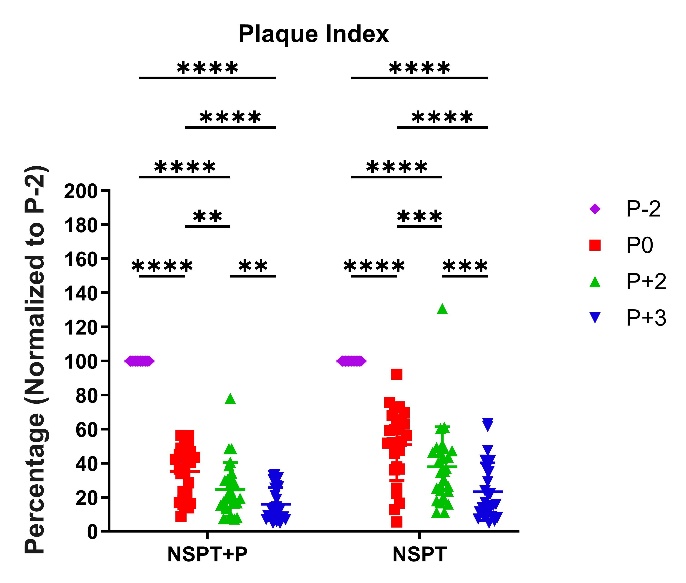


**C**

**D**

**Supplementary Figure 2.** Clinical indicators of periodontitis in the NSPT and *S. Salivarius* M18 group (NSPT+P) and the NSPT and placebo group (NSPT) at the time points P0, P+2, and P+3, presented in percentages normalized to the baseline at the time point P-2 and compared among the different time points within each group. A) Pocket probing depth greater than 4 mm. Significant reduction was observed progressively at each time point within each of the two groups. B) Clinical attachment loss. Compared to the baseline at P-2, significant reduction was observed at P+2 and P+3 in the NSPT+P group and at P0, P+2, and P+3 in the NSPT group. C) Bleeding on probing. Significant reduction was observed progressively at each time point within each of the two groups. D) Plaque index. Significant reduction was observed progressively at each time point within each of the two groups. (**** P ≤ 0.0001, *** P ≤ 0.001, ** P ≤ 0.01, * P ≤ 0.05; two-way ANOVA, Tukey’s multiple comparisons test).
